# Brain-Derived Neurotrophic Factor in Multiple Sclerosis Disability: A Prospective Study

**DOI:** 10.3390/brainsci14030243

**Published:** 2024-02-29

**Authors:** Vitalie Vacaras, Andreea-Cristina Paraschiv, Silvina Iluț, Cristiana Vacaras, Cristina Nistor, Gheorghe-Eduard Marin, Andra Maria Schiopu, Dorian-Traian Nistor, Ștefan Cristian Vesa, Dafin Fior Mureșanu

**Affiliations:** 1 Neurology Department, Cluj Emergency County Hospital, 400012 Cluj-Napoca, Romania; vvacaras@umfcluj.ro (V.V.); silvina.ilut@yahoo.com (S.I.); cristinapinzaru@yahoo.com (C.N.); dafinm@ssnn.ro (D.F.M.); 2 Department of Neurosciences, Faculty of Medicine, Iuliu Hațieganu University of Medicine and Pharmacy, 400012 Cluj-Napoca, Romania; cristina.andreea.paraschiv@gmail.com; 3 Faculty of Medicine, Iuliu Hațieganu University of Medicine and Pharmacy, 400349 Cluj-Napoca, Romania; edymarin53@gmail.com (G.-E.M.); schiopuandra.22@gmail.com (A.M.S.); 4 Department of Oncology and Radiotherapy, Faculty of Medicine, Iuliu Hațieganu University of Medicine and Pharmacy, 400349 Cluj-Napoca, Romania; nistor.dorian@yahoo.com; 5 Department of Pharmacology, Toxicology and Clinical Pharmacology, Faculty of Medicine, Iuliu Haţieganu University of Medicine and Pharmacy, 400349 Cluj-Napoca, Romania; stefanvesa@gmail.com

**Keywords:** demyelinating disease, multiple sclerosis, brain-derived neurotrophic factor, BDNF, interferons, teriflunomide, immunomodulating agents, neurologic manifestations, magnetic resonance imaging

## Abstract

Multiple sclerosis (MS) is a demyelinating central nervous system disease that leads to neurological disability. Brain-derived neurotrophic factors (BDNFs) are neurotrophins involved in neurodegenerative disorders. This study analysed the relationship between serum BDNF, neurological disability and different MS treatments. We included 63 people with MS (PwMS), with relapsing-remitting MS or clinically isolated syndrome, and 16 healthy controls (HCs). We analysed the serum levels of BDNF and MS specific disability tests (Expanded Disability Status Scale, timed 25-foot walk test, nine-hole peg test), at baseline (V0) and after one year of interferon beta1a or teriflunomide treatment (V1). Baseline BDNF values were not different between the PwMS and HCs (*p* = 0.85). The BDNF levels were higher in PwMS vs. HCs after treatment (*p* = 0.003). BDNF was not related to last-year relapses or by the disease duration (all *p* > 0.05). The overall values for the PwMS decreased after one year (*p* < 0.001). Both treatments implied a similar reduction. BDNF was not related to neurological disability (*p* > 0.05). BDNF values were not influenced by the lesion burden, active lesions, or new lesions on MRI (*p* > 0.05). In our cohort, the PwMS had higher BDNF levels compared to the HCs after one year of treatment. BDNF was not related to clinical or paraclinical disease severity signs.

## 1. Introduction

Multiple sclerosis (MS) is an autoimmune demyelinating disease of the central nervous system, and it represents the most common non-traumatic disease that causes neurological disability in young people [1]. It is estimated that 2.8 million people worldwide live with MS, with an increasing prevalence in every area of the world reaching 35.9 per 100,000 people in 2020. The general pooled incidence rate is 2.1 per 100,000 persons/year. The mean age of diagnosis is 32 years, with a higher prevalence in females than males (2:1 ratio) [2].

The pathology has two stages: inflammation responsible for relapsing-remitting disease and late neurodegeneration in the progressive stages. It is mostly characterised by the appearance of perivenular inflammatory lesions with immune cells including T-lymphocytes, B cells and plasma cells, which leads to the demyelination process. Further on, irreversible axonal damage can occur [1]. The clinical features of the disease are classified into clinically isolated syndrome (CIS), relapsing–remitting form (RRMS), primary progressive (PPMS) and secondary progressive MS (SPMS). Patients typically present with optic neuritis, spinal cord and brainstem syndromes, with a large variety of symptoms. Diagnosis is based on a combination of clinical factors, imagistic lesions and, in some cases, lumbar puncture [3,4]. Disease-modifying therapies (DMTs) include immunomodulatory drugs, such as interferon beta1a (IFN-β 1a) and teriflunomide (TER), which aim to reduce long-term disability [1]. The mechanisms of the disease are complex. It is considered that based on a genetic susceptibility, some environmental factors such as vitamin D exposure, Ebstein–Barr infection and gut microbiome alterations contribute to the disease onset [1].

Neurotrophins are possible mediators for immune-related diseases of the central nervous system (CNS). Brain-derived neurotrophic factor (BDNF) is a common neurotrophic component of the CNS, mostly produced in the brain, including neural growth factor and neurotrophins 3 and 4 [5]. BDNF has an important role in the development of the nervous system, as well as in supporting neuron survival, neurogenesis, neuroplasticity and neurotransmission. It is mostly distributed in the cortical areas, hippocampus and visual cortex. Multiple factors have been shown to increase BDNF levels: physical activity, light and seasonal variances [6].

The precursor proBDNF is converted into mature mBDNF. BDNF binds to two plasma membrane receptors: tropomyosin kinase B (TrkB) and p75 neurotrophin. Mature BDNF has an affinity for the TrkB receptor, which promotes cell survival. The BDNF/TrkB complex is internalised into the neurons and induces a signalling cascade, including phospholipase C, mitogen-activated protein kinase, protein kinase B and guanosine triphosphate hydrolases. These pathways are involved in maintaining synaptic integrity and an antiapoptotic effect. On the other hand, the precursor component mostly associates with the p75 neurotrophin receptor, inducing apoptosis [7]. The insufficient transformation of proBDNF into mBDNF due to abnormal proteolytic cleavage leads to a change in BDNF levels in the peripheral blood and CNS, and it might be involved in the pathogenesis of various diseases, such as depression, Parkinson’s disease, amyotrophic lateral sclerosis and multiple sclerosis [8].

Various studies have analysed BDNF levels as a potential biomarker for MS in order to establish a possible correlation with the neurodegeneration process. Some studies observed that serum BDNF levels are generally reduced in people with MS (PwMS) compared to healthy controls [9,10,11,12]. A 3.5-fold decrease in the gene expression of BDNF corresponded to a 1.5-fold downregulation in the BDNF plasma concentration [13]. The potential loss in neuroprotection is due to a decrease in BDNF synthesis in CNS [14]. Others have found that BDNF levels are significantly higher in stable or active RRMS but reduced in SPMS or PPMS [15]. Another study observed the same BDNF to increase in RRMS individuals compared to controls [16]. Some studies demonstrated a significant increase in serum BDNF during relapses [17,18], which sustain the hypothesis that neurotrophic factors promote remyelination [19]. Immune cells positive for BDNF were found in the active lesions in MS, as seen on immunohistochemistry analyses, while chronic inactive lesions have fewer BDNF cells. In the progressive form of the disease, we find a deficit in the antegrade transport of the axons and a reduced BDNF beneficial role. The Val66Met polymorphism in the BDNF gene was associated with an altered BDNF activity, leading to hippocampal and memory deficits. It was more frequently encountered among MS participants, where the Val/Met polymorphism is associated with higher BDNF levels, better cognitive performance and better grey matter conservation [20]. However, in most studies, there was no difference in BDNF values between PwMS and healthy controls [21,22].

The effect of different immunomodulatory drugs on BDNF levels are unclear. Therefore, various studies have searched for a relationship between neurotrophins and MS specific treatments. Interferons have been the most studied from this category, with inconsistent results. Some studies stated a significant effect of IFN-β1a on the BDNF level in MS patients. In an observational study from 2013, BDNF levels significantly increased at 3 and 6 months after treatment, but they gradually decreased at 12 months of follow-up [23]. Other authors outlined that neurotrophic factors are correlated with higher inflammatory activity and higher white matter volume [24]. In some papers, IFN-β1a-treated patients presented lower BDNF concentrations compared to untreated subjects [25]. Lalive et al. demonstrated a decreased bioactive BDNF form in untreated RRMS compared to controls, while there was an increase in this active form for IFN-treated participants [22]. In a recent study performed on 45 RRMS patients and 45 healthy controls, they found no difference in BDNF levels for IFN-treated and untreated patients [26]. In another study performed on 50 PwMS, the BDNF expression did not change after one year of first-line treatment with IFN or glatiramer acetate [12]. The effect of teriflunomide on BDNF secretion is less studied in the literature.

As an important factor in neuroplasticity and neurorecovery, many have studied the relationship between disability and BDNF. A study explored the possible correlation between serum and cerebrospinal fluid (CSF) BDNF levels and disability outcomes. The results stated that increased BDNF preceded neurological and cognitive improvement in PwMS. While participants with an improvement in neurological disability had higher serum BDNF levels compared to those without, increased CSF BDNF baseline levels were related to cognitive improvement. Also, significant correlations were found between BDNF concentrations and the EDSS (Expanded Disability Status Scale) score, as a standardised measure for MS disability [27].

Our hypothesis was that the serum BDNF levels are correlated with neurological disability in MS and different specific treatments can influence its concentration.

This study aimed to analyse the variations in the serum BDNF levels of MS patients and their correlation with the disease severity. We identified BDNF variability in time, compared the values of MS patients with healthy controls, studied the effect of different treatments on BDNF and correlated BDNF with other variables, such as disease duration, relapses, brain MRI lesions and neurological disability tests.

## 2. Materials and Methods

### 2.1. Subjects

Our observational, longitudinal, prospective, and case-control-type study included 63 PwMS from the Neurology Department in The Emergency Hospital Cluj-Napoca, Romania, and 16 healthy controls (HC); data were collected from January 2019 to May 2020. The study was conducted according to the Helsinki Declaration and ethical authorisation was approved by The Emergency Hospital Cluj-Napoca, number 2394/28.01.2020. Each subject signed an informed consent form.

The inclusion criteria were adult participants, diagnosed with MS according to the 2017 Revised McDonald Diagnosis Criteria [3], either RRMS or CIS, with an Expanded Disability Status Scale (EDSS) score of a maximum of 5 points and not treated with any DMT for at least one year before study enrolment. We excluded progressive forms of the disease, unclear diagnoses, and patients with incomplete medical histories or examinations. The control group (HC) was formed of healthy individuals without any known pathologies or chronic medication. We enrolled PwMS who have been recently diagnosed with MS and that were planning to receive a first-line DMT, either IFN-β1a 30 mcg intramuscular once a week (group G(IFN)) or TER 14 mg orally, daily (group G(TER)) (based on their personal preferences or disease characteristics). In some cases, we also included patients with a higher disease duration, but after a wash-out period of at least one year of any DMT. The HC were healthy participants who accepted to participate in this study and that matched the demographics of the patients in terms of characteristics such as age and sex.

### 2.2. Instruments and Procedure

We performed two follow-up visits: the first one, at the study onset, V0, before receiving any treatment for the case group, and the second visit, V1, at least one year after the first visit. Each visit included a blood sample analysis, demographic and disease characteristic questionnaires, and, for PwMS, specific tests for evaluating different disease severity aspects: the Expanded Disability Status Scale (EDSS), timed 25-foot walk test (T25FW), 9-hole peg test (9HPT) and a mini-mental state examination (MMSE). Out of 63 PwMS, we obtained complete MRI data for 38 participants. Two contrast brain MRIs were performed at each visit. In each MRI scan, we quantified the MRI total lesion burden and the total amount of active lesions, while in the second MRI, we also searched for new or enlarging T2 lesions. We classified the lesion burden in one of the three following groups, according to the approximative lesion number: less than 2 lesions, between 2 and 9 lesions and more than 9 lesions.

The EDSS is an objective standardised method of evaluating the degree of neurological disability in MS, with scores ranging from 0 to 10. It evaluates several functional systems: the pyramidal, cerebellar, brain stem, sensory, bladder, visual and mental systems, with each system being graded on a specific functional score (FS) from 0 to 5 or 6, according to the pathological findings upon neurological examination. Binding each FS with the ambulation capacity of the patient (their ability to walk a measured distance and the analysis of any external aid), we obtained the final EDSS score. A score of 0 points is for a complete normal neurological examination, a score of 1 or 1.5 p stands for the lack of any disability but minimal signs on FS examinations, an EDSS score of 2–2.5 p means minimal disability in at least one FS, and a score of 3–3.5 p stands for a moderate disability but still a fully ambulatory patient. A score higher than 4 points represents gait impairment. Scores ranging from 4 to 5.5 reflect the capacity to walk without aid but on a limited specified distance. A 6.0 p score outlines permanent unilateral assistance; a 6.5 score, bilateral aid; and 7–7.5 p, permanent need for a wheelchair. A bed-restricted patient has an 8 p score, while the maximum of 10 p reflects death due to MS [28].

T25FW is a quantitative method for gait evaluation in PwMS. It is an easy-to-administer, short format walking test, commonly used in clinical practice and useful for evaluating walking disability. This test correlates well with other measures of walking ability. It records the time (in seconds) an individual needs to walk 25 feet as quickly as possible, using an assistive device if necessary. The time needed for walking the measured distance is recorded twice, and then the average mean between the two examinations is registered. It is usually considered that a 20–25% decline in walking speed may be a clinically meaningful threshold for defining gait worsening in MS clinical trials but also in monitoring patients in a clinical setting [29].

9HPT is a test quantifying the functionality of upper extremities (arm and hand), where the patients use their coordination. Both dominant (9HPT-D) and non-dominant (9HPT-ND) hands are tested twice, on a solid table and with a stable position of the 9-HPT apparatus. We quantify the time (seconds) the participant needs to pick-up each of 9 pegs at a time, using one hand only, and put them into the holes as quickly as they can until all the holes are filled. Then, without pausing, the participant removes the pegs one at a time and returns them to the container as quickly as possible [30].

MMSE is a simplified screening test for cognitive deficiencies, accounting for temporospatial orientation ability, attention, memory and language. It includes eleven questions, and it is therefore practical to use in clinical routine. It mostly concentrates on cognitive aspects of mental function, and it excludes issues regarding mood or the form of thinking. It is divided in two sections, the first of which requires only verbal responses and covers orientation, memory and attention and the second part considers the ability to name, follow commands and copy a drawing. The maximum score is 30 points. Values above 34 p indicate normal cognitive function; 19–23 p, mild deficits; 10–18 p, moderate deficits; and below 9 points indicates severe cognitive impairment. The test is not timed but it has a limitation for participants with visual and verbal deficits [31].

Blood samples collected from participants were centrifuged and preserved at minus 20 degrees Celsius until data processing. We used ELISA kits to analyse the serum, as specified by the manufacturer’s instructions, Human BDNF ELISA Kit, Catalog Number EH42RB (96 tests), Thermo Fisher Scientific Inc., Waltham, MA, USA.

### 2.3. Statistical Analysis

Statistical analyses were performed using Statistical Package for Social Sciences (SPSS Inc., Chicago, IL, USA), version 20.0.0. The statistical significance cut-off value of *p* was lower than 0.05 for all the statistical tests utilised. For graphical outputs, we used Microsoft Excel (2016). The value distribution was assessed for all quantitative variables using the Kolmogorov–Smirnov Test of Normality with the Lilliefors Significance Correction.

Following this assessment, statistical tests were chosen based on the results from the Kolmogorov–Smirnov test, as outlined in Table S1 from the Supplementary Materials. For variables that follow a normal distribution, a parametric test such as an Independent Sample T-Test and one-way ANOVA were employed to compare means and standard deviations. An Independent Sample T-Test compared the BDNF values between MS and HC at baseline and after treatment, BDNF variability in time, BDNF and relapses or MRI lesions. ANOVA tests were used for analysing BDNF means from the 3 disease-type groups. Additionally, we performed a two-way repeated ANOVA test for BDNF variability related to different aspects. A Paired Samples T-Test was used to compare the values of a variable between two timepoints for the same group (the two BDNF values from the same group). For variables that did not follow a normal distribution, non-parametric variants of the aforementioned tests were used, mainly the Independent Sample Mann–Whitney U Test and Spearman Correlation, in order to compare median differences or assess monotonic relations, respectively. An Independent Sample Mann–Whitney U Test assessed the functional test results between groups. Spearman Correlation was used mainly for the monotonic relationship between BDNF values and disability tests or disease duration. The Related-Sample Wilcoxon Signed-Rank Test was used to compare the values of a non-normally distributed variable between two points in time for the same group. The McNemar Test was used to assess changes in qualitative variables between two moments in time for the same group, as in our case for the differences between MRI scans parameters.

We performed an optimal sample size calculation to limit a type 2 error, beta. Firstly, at a global level, with an MS rate of 0.0359% (according to the literature data [2]), a 1.25% margin of error and a 99.99% CI (confidence interval), the minimum sample size for PwMS would be 45. Considering Transylvania as the representative population for our study [32], a 0.5% margin of error and a 95% CI, the minimum sample size is 47 participants with MS.

The results of the test have been reported as the value of *p* and specific metrics depending on the distribution (mean and standard deviation for parametric tests, and median and interquartile range for non-parametric tests).

## 3. Results

### 3.1. Subjects’ Characteristics

We included 56 participants diagnosed with MS and 7 individuals diagnosed with CIS. The mean age of the PwMS was 31 years, ranging from 19 to 59, with a female predominance (65%), SMRR majority (88%) and a mean EDSS score of 1.81 points, ranging from 0 to 4 points. The HC group had a mean age of 31, in a range of 20–50. Other demographic and clinical features can be seen in Table 1.

### 3.2. BDNF Values at Baseline and after Treatment

The mean BDNF baseline value for the PwMS was 36.29 ng/mL and 36.21 ng/mL for the HC. The boxplot in Figure S1 from the Supplementary Materials outlines the BDNF similarities at baseline between MS and the HC. As detailed in Table 2, there were no statistically significant BDNF differences between the PwMS and HC at baseline or between RRMS, CIS and HC. The G(TER) group had higher BDNF values compared to the group G(IFN). Baseline BDNF values were not related to the living environment (background) or sex. Considering the September–February period as the cold season and March–August as the hot season, there was no seasonal difference in BDNF (*p* = 0.35). The baseline BDNF values were not significantly related to the disease duration or the presence of any relapse in the past year.

Using BDNF at baseline as the dependent variable, and MS type (RRMS, CIS or HC), MS diagnosis (PwMS or HC), treatment (IFN-β1a or TER), background (urban or rural), sex (male or female) and season (cold or warm) as fixed factors for a two-way ANOVA (Univariate Analysis of Variance), it returned a corrected model with a *p* = 0.722, suggesting that none of the fixed factors or their interactions contributed to the values of BDNF at baseline significantly.

**Figure 1:**
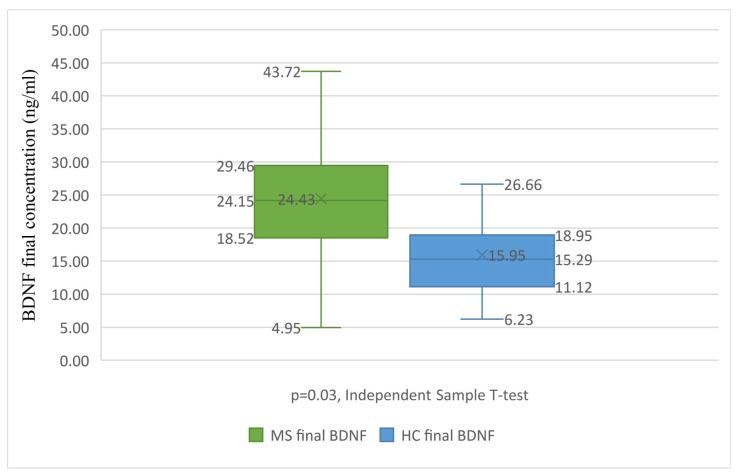
BDNF differences between treated MS and the HC. Boxplot graph presenting the differences between BDNF levels after the treatment of PwMS (in green) and HC (in blue). The Independent Sample T-Test reveals statistically significant differences between the groups (*p* = 0.003), with the HC group having significantly lower BDNF values. BDNF, brain-derived neurotrophic factor; HC, healthy control; MS, multiple sclerosis. X, mean value; lower box limit, 25% quartile value; central line, median value; upper box limit, 75% quartile value; lower whisker, minimum value; upper whisker, maximum value.

When studying BDNF at the second follow-up visit, after one year of treatment, the mean BDNF for the PwMS was 24.43 ng/mL and 15.95 ng/mL for the HC. The PwMS had significantly higher values of BDNF (7.68 units) compared to the HC (*p* = 0.03, Independent Sample T-Test), as emphasised in Figure 1. RRMS had 8.27 units higher BDNF values compared to the HC. After one year of treatment, G(TER) had greater BDNF values compared to the HC. Compared to G(IFN), G(TER) also had greater BDNF values, but when applying corrections due to this baseline difference between groups, the difference is not significant anymore. We also found no difference when considering background or sex. The second BDNF value was not related to the presence of any clinical relapse in the past year of follow-up, either for G(IFN) or G(TER).

Using BDNF values from the second follow-up visit as the dependent variable, and the same fixed factors as those previously mentioned for a two-way ANOVA, it returned a corrected model with a *p* = 0.003 and an observed power of 0.993, suggesting that some of the fixed factors or their interactions significantly contributed to the BDNF values.

The *p* values, partial eta squared parameters and observed power for the significant fixed factors and their interactions can be found in Table 3.

The means and standard deviations of BDNF values grouped based on fixed factors or their interactions can be found in Tables S2–S5 from the Supplementary Materials.

### 3.3. BDNF Differences in Time

**Figure 2:**
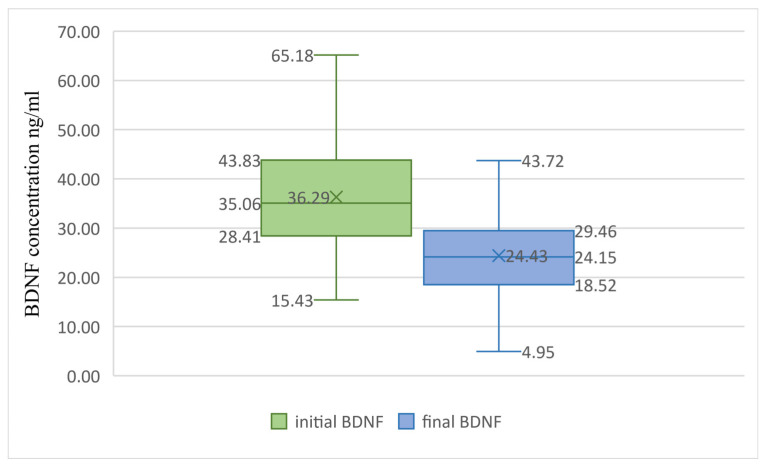
BDNF differences in time for PwMS. Boxplot graph presenting the differences between baseline BDNF levels (in green) and BDNF levels after treatment (in blue). The Paired Sample T-Test reveals statistically significant differences between the groups (*p* = 0.001), with the final BDNF values being significantly lower. BDNF, brain-derived neurotrophic factor. X, mean value; lower box limit, 25% quartile value; central line, median value; upper box limit, 75% quartile value; lower whisker, minimum value; upper whisker, maximum value.

We tested the differences between V0 and V1. For all our subjects, the BDNF levels were significantly lower (13.3 units) at the second follow-up visit compared to baseline (*p* = 0.001, Paired Sample T-Test). For all the PwMS, there was a reduction of 11.86 units in BDNF at the second visit (*p* < 0.001), as outlined in Figure 2.

We analysed if one year of DMT influenced the BDNF values. With a Paired Sample T-Test, we found similar reductions in BDNF for both teriflunomide (12.7 units, *p* < 0.001) and interferon beta1a (10.99 units, *p* < 0.001).

There was no connection between BDNF and the season the blood sample was collected in, for all subjects (*p* = 0.07, Related Sample McNemar Test) or the PwMS (*p* = 1.00).

We also quantified the differences between the first and the second values for the BDNF for each subject, to reflect the variability between samples in time. We defined a variable delta V as the difference between the BDNF concentration at V0 and V1 (positive numerical value). There was a significant difference in variability between the PwMS and HC (*p* = 0.02, Independent Sample T-Test), with the HC group having a variation between the follow-up and baseline of 7.11 units more than that of the PwMS. A difference was also found between RRMS, CIS, and HC (*p* = 0.04, ANOVA test); thus, we performed post-hoc tests to identify it more specifically. The variations in BDNF for the HC group were greater than the variations in RRMS (*p* = 0.03) or CIS (*p* = 0.02).

There was no difference in delta V for interferon when compared to teriflunomide (*p* = 0.54, Independent Sample T-Test), which confirms that the V1 differences between these two groups were due to their starting values being different at V0.

The delta V variable was not correlated to any clinical relapse in the follow-up period (*p* = 0.301, Independent Sample T-Test) in the case of all the PwMS, both treated with interferon (*p* = 0.667, Independent Sample T-Test) or teriflunomide (*p* = 0.109, Independent Sample T-Test).

### 3.4. Clinical and Paraclinical MS Tests

To identify if there is any relationship between BDNF and the outcomes from the disability functional tests, we performed Spearman Correlations between all variables, as outlined in Table 4. The scores were not correlated with BDNF (all *p* > 0.05), except for 9HPT-D, with a significant *p* of 0.01 and a correlation coefficient of −0.32.

We studied the influence of having received treatment on functional test scores. T25FW scores at the second visit were significantly decreased (0.53 s) for interferon beta1a compared to the teriflunomide group (*p* = 0.04, Independent Sample Mann–Whitney U Test). All other scores were similar between treatment groups for the second evaluation (*p* > 0.05).

The differences between the two MRIs were not statistically significant in terms of the lesion burden (*p* = 1, McNemar Test) or the number of active lesions (*p* = 0.057, McNemar Test).

The baseline BDNF values were not influenced by the lesion burden (*p* = 0.324, Independent Sample T-Test) or the presence of any active lesions (*p* = 0.536, Independent Sample T-Test). Also, the BDNF levels from the second visit were not influenced by the lesion burden (*p* = 0.13, Independent Sample T-Test) or the presence of any active lesions (*p* = 0.475, Independent Sample T-Test). The Independent Sample T-Tests outline that the occurrence of new lesions in MRI scans did not significantly influence the BDNF concentration from V0 (*p* = 0.641), V1 (*p* = 0.297) or delta BDNF (*p* = 0.19).

## 4. Discussion

Considered the most common cause of disabling non-traumatic disease in young adults, MS is an inflammatory neurodegenerative disease, with complex and various unknown pathophysiological factors [1]. Its growing incidence has led to the need to identify biomarkers for more personalised healthcare. A reliable biomarker should estimate the disease susceptibility, enable early diagnosis, encourage new therapeutic pathways or predict the disease severity [20]. BDNF is a pivotal neurotrophin for the CNS, as it helps maintain the structural integrity of neurons and influences how they function and if they survive. It is produced by neurons, oligodendrocytes, platelets, immune cells and active muscles [20]. BDNF is thought to cross the blood–brain barrier (BBB) in both directions. Thus, peripheral BDNF levels mostly reflect the CNS’ amount [20].

In this study, we analysed the serum levels of BDNF in MS as a potential biomarker for the disease’s severity. Also, we searched for the effect of first-line treatments on peripheral neurotrophic factors since there are contradictory and limited data in the literature. Studies focused on teriflunomide related to BDNF are limited.

### 4.1. Serum BDNF Values in PwMS

Most studies on BDNF in MS have contradictory results, possibly due to different sample analyses (blood or CSF) and laboratory methods being used and the inability to differentiate mature BDNF from proBDNF [8]. Although the results are controversial, showing both higher and lower levels compared to a HC, numerous studies outlined that peripheral BDNF levels are decreased in MS patients compared to the HC, as a result of decreased neuroprotection [8,13,20]. In a review including 689 PwMS, BDNF was found to be less present compared to in the healthy population, and the difference was obtained also through serum analysis [10]. In a study with 259 PwMS, baseline BDNF levels were also reduced, although the difference was insufficient to be relevant in any disease decision process [33]. By comparing the gene expression related to the peripheric protein levels from an MS population, a study found that a decrease in the BDNF gene expression corresponds to a downregulation in the final plasmatic concentration [13].

In our cohort, the baseline BNDF levels of PwMS were similar to those of the HC at study onset (*p* = 0.85). After one year of treatment, MS patients had higher BDNF values compared to the HC (*p* = 0.03), which is contradictory to most of the literature studies. Also, RRMS individuals had higher values compared to the HC. Our patients had been diagnosed with the disease not long before the study onset, and, in the early stages of the disease, we generally encounter more relapses, as the inflammatory state stimulates BDNF expression as a repair mechanism. Also, we had an imbalanced case-control ratio that could influence the statistical significance of our results.

### 4.2. BDNF Concentrations Related to the Clinical Disease Activity

However, neurotrophins are generally more present in MS inflammatory lesions, because of the BDNF role in the process of remyelination in an acute inflammatory lesion [20]. The presence of BDNF is enhanced by immune cells in areas of ongoing active demyelination at the peripheral sites of the lesions in the disease’s early stages, and it is reduced in chronic lesions. Thus, peripheral BDNF is generally higher in participants with active demyelinating lesions during relapses [4,17,18,34]. Neurotrophic factors are correlated with higher inflammatory activity [24].

Disease duration and the occurrence of any relapse one year prior to our blood tests did not influence the BDNF levels in our cohort. This would be explained by the presence of a remission phase in most our patients. In various studies, we find a positive correlation between BDNF and different disease characteristics only in the relapse phase, with no differences in the remission phase [34].

### 4.3. BDNF Concentrations Related to the Paraclinical Disease Activity

As a neuroprotective agent, BDNF has a role in neural repair [6]. We hypothesised that MS lesions on brain MRI could be related to the serum level of BDNF, as a response to injury. In our study, we found no correlation between the BDNF concentrations and the number of total lesions or active lesions visible on T2 weighted images. The literature results are contradictory on this aspect, with some studies suggesting a positive correlation between BDNF and disease activity, expressed by gadolinium-enhanced lesions on brain MRI or by the number of T2 infratentorial lesions in relapse. However, in the remission phase, MRI lesions are not related to BDNF levels [34]. Other studies found no relationship between BDNF and MRI characteristics [33].

### 4.4. Treatment Influence on BDNF Levels

The first treatment category of DMTs includes immunomodulatory drugs, such as interferon beta1a and teriflunomide [1,35]. The literature research has contradictory results on the effects of MS therapies on BDNF levels. The results vary according to the studies’ characteristics and the specific analysed treatments. Some studies report a significant increase in BDNF after IFN-β1a treatment, but a gradually decrease until 12 months [23]. Others have stated a decrease in the bioactive BDNF form in untreated PwMS compared to the controls, while there is an increase in this active form for IFN-treated participants [22] Shajarian et al. found no difference between the BDNF levels for IFN-treated and untreated patients [26], while Karmand et al. stated that IFN-β1a-treated patients presented even lower BDNF concentrations compared to untreated subjects [25].

When studying how treatment influenced BDNF levels in our cohorts, there was a reduction in BDNF for both the teriflunomide and interferon groups, with a similar unit level. Therefore, the decrease in BDNF in the second evaluation cannot be attributed to the treatment.

### 4.5. Disability Outcomes Related to BDNF

In our study, BDNF was not correlated to a neurological impairment, as quantified by functional tests, such as the EDSS, T25FW, and 9HPT. The positive correlation between the dominant hand in the 9HPT and BDNF was isolated and not replicated in all situations; thus, it is most probably not significant. In the literature, some studies have also found no association between BDNF and EDSS [33]. However, an EDSS change can be related to serum BDNF in other papers, since subjects with disability improvements measured by the EDSS score had increased BDNF levels compared to those without these improvements [27].

### 4.6. Other Factors That Influence BDNF in PwMS

Although the literature indicates seasonal variations in BDNF, with increased values in sunlight periods [6], our study revealed no correlations between BDNF and the sample collecting season for the first sample. However, the two-way ANOVA test performed for the follow-up visit revealed that BDNF might be related to seasonal exposure, with a *p* value of 0.04.

With an important role in neuronal plasticity, neurotrophic factors are aimed to enhance the regenerative brain function, and they are studied as a potential therapy for neurodegenerative diseases. Different strategies that aim to enhance BDNF levels in the MS population need to be studied further [36].

Although various studied factors were not related to BDNF levels, we found for the second sample that the combination of some fixed factors can influence BDNF, such as MS type and sex or treatment combined with background and sex. These associations need further studies for accuracy.

All the inconclusive results of this study may be due to the relatively short time of a follow-up of one year, as a longer period of time might be needed to observe significant changes.

## 5. Limitations and Strengths

The strengths of this study reside on a dynamic follow-up of serum BDNF concentrations in an MS population over one year, analysing different variables such as specific treatments and disability outcomes. In the literature, we find less studies on teriflunomide and BDNF, and also, we usually encounter only EDSS scores for disease progression and not other tests, such as the T25FW and 9HPT.

The first limitation of this study is the number of healthy subjects being smaller than the patient group, with a case-control ratio of 4:1, due to the fact that our main objective was to study BDNF in an MS population, with its dynamic changes and the treatment influence. The comparison between the BNDF values of the PwMS and HC was a secondary objective, since our main focus was on the longitudinal analysis of the MS characteristics. This imbalance does not affect the analysis of the MS cohort; however, it can reduce the statistical accuracy of or case-control comparisons. Secondly, BDNF values are highly variable according to multiple co-founders, thus making their variations difficult to interpret due to possible bias factors. Another possible bias source is the lack of a randomisation process. Also, we mention the possible bias of the 25 participants lost during the imagistic analysis, as we did not obtain complete MRI data for all subjects.

## 6. Clinical Implications/Future Directions

BDNF is regarded as a potential biomarker for monitoring the progression of different neurodegenerative pathologies, but it can also become a target for treatment as well. Based on the findings that BDNF can cross the blood–brain barrier from periphery to the central nervous system, numerous therapeutic strategies have emerged. So far, experimental therapies on animals show promising results, with increases in neurogenesis and normal neural activity after intrathecal infusion in rats [5].

Various techniques have been used to deliver neurotrophic factors in the brain, of which the most common is direct intracerebroventricular infusion. Another studied direction would be viral vector-mediated gene delivery methods. Also, different cell types have been used to deliver neurotrophins to the specific sites, with positive results so far [37].

Future studies might improve the BDNF analysing methods in laboratories, thus making it a more reliable biomarker for disease severity and a potential therapeutic target for complex neurological disorders.

## 7. Conclusions

Our study revealed higher BDNF values for MS patients after one year of treatment. Overall, the values decreased over time. BDNF was not associated with the disease’ clinical or paraclinical outcomes, such as in terms of the EDSS, T25FW, 9HPT, MMSE or MRI lesions. The suitability of BDNF as a biomarker for the disease severity or its neural repair effects in MS needs to be studied further.

## Figures and Tables

**Table 1:** Subjects’ demographic and clinical characteristics.

Characteristics	PwMS (*n* = 63)	HC (*n* = 16)
Sex, *n* (%)		
males	22 (34.92)	5 (31.2)
females	41 (65.07)	11 (68.7)
Age, median (SD), y	33 (9.82)	28.5 (8.88)
MS type, *n* (%)		
RRMS	56 (88.88)	
CIS	7 (11.11)	
EDSS score, mean (SD)	1.81 (0.83)	
MMSE score, mean (SD)	29.33 (1.02)	
Treatment, *n* (%)		
Interferon beta1a	31 (49.1)	
Teriflunomide	32 (50.7)	
Disease duration, mean (SD), y	4.86 (3.8)	
Number of relapses, mean (SD)	2.55 (2.06)	

Abbreviations: MS = multiple sclerosis; PwMS = people with multiple sclerosis; HC = healthy control, *n* = number of participants, SD = standard deviation, y = years, RRMS = relapsing–remitting MS; CIS = clinically isolated syndrome; EDSS = Expanded Disability Status Scale; MMSE = mini-mental state examination.

**Table 2:** Differences in BDNF values at baseline and after treatment.

BDNF Values Comparison	V0	V1	Statistical Tests
PwMS vs. HC	*p* = 0.85	*p* = 0.03 *	Independent Sample T-Test
RRMS vs. CIS vs. HC	*p* = 0.07	*p* = 0.004 *	ANOVA test
RRMS vs. HC	*p* > 0.05	*p* = 0.004 *	Bonferroni test
G(TER) vs. G(IFN)	*p* = 0.02 *	*p* = 0.04 uncorrected *p* = 0.45 corrected	Independent Sample T-Test
G(TER) vs. HC	*p* > 0.05	*p* = 0.001 *	ANOVA and Bonferroni test
rural vs. urban background	*p* = 0.06	*p* = 0.55	Independent Sample T-Test
female vs. male gender	*p* = 0.06	*p* = 0.23	Independent Sample T-Test
presence of any relapse in the last year	*p* = 0.82	*p* = 0.13 *p* = 0.27 for G(IFN) *p* = 0.32 for G(TER)	Independent Sample T-Test
disease duration	*p* = 0.27	-	Spearman Correlation

* statistically significant values. Abbreviations; PwMS = people with multiple sclerosis; HC = healthy control, RRMS = relapsing–remitting multiple sclerosis; CIS = clinically isolated syndrome, TER = teriflunomide, IFN = interferon.

**Table 3:** BNDF interactions with fixed factors, after one year of follow-up.

Fixed Factor	*p* Value	Partial Eta Squared	Observed Power
Season	0.047 *	0.0722	0.514
MS type × Sex	0.019 *	0.0994	0.661
Treatment × Background	0.019 *	0.0992	0.659
Treatment × Background × Sex	0.001 *	0.1788	0.915
Treatment × Background × Season	0.018 *	0.1008	0.667

* statistically significant values, two-way ANOVA (Univariate Analysis of Variance).

**Table 4:** Spearman Correlations between BDNF values and functional MS tests.

Tests Scores	*p* Value (V0)	*p* Value (V1)
EDSS	0.52	0.42
T25FW	0.79	0.21
9HPT-D	0.31	0.01 * (r = −0.32)
9HPT-ND	0.19	0.09
MMSE	0.17	0.25

Abbreviations: r = correlation coefficient; * = statistically significant

## Data Availability

The data and other information of this study are available from the corresponding author upon request, The data are not publicly available due to patients’ privacy (identification details that must not be made public).

## Supplementary Materials

The following supporting information can be downloaded at: https://www.mdpi.com/article/10.3390/brainsci14030243/s1, Table S1: Statistical significance of Kolmogorov-Smirnov test of normality for tested. Table S2: Mean and standard deviation of BDNF for Season as a fixed factor. Table S3: Mean and standard deviation of BDNF for MS Type and gender as fixed factors and their combined influence. Table S4: Mean and standard deviation of BDNF for Treatment, background, and gender as fixed factors and their combined influence. Table S5: Mean and standard deviation of BDNF for Treatment, background, and season as fixed factors and their combined influence. Figure S1: Baseline BDNF differences between MS and HC.
