# Supplementary material for: Brain-Derived Neurotrophic Factor in Multiple Sclerosis Disability: A Prospective Study
Source: Brain Sci. 2024 Feb 29;14(3):243. doi: 10.3390/brainsci14030243 (PMC10968117; doi:10.3390/brainsci14030243)
Supplement: Supplementary file 1 [file brainsci-14-00243-s001.zip › brainsci-2880074-supplementary.pdf]

**Table S1*.** Statistical significance of Kolmogorov-Smirnov test of normality for tested

| Variable | p Value |
|---|---|
| BDNF baseline | 0.200* |
| BDNF follow-up | 0.200* |
| Delta V | 0.200* |
| Age | <0.001 |
| Initial Average T25FW | 0.042 |
| Initial Average 9HPT dominant hand | <0.001 |
| Initial Average 9HPT non-dominant hand | <0.001 |
| Final Average T25FW | <0.001 |
| Final Average 9HPT dominant hand | <0.001 |
| Final Average 9HPT non-dominant hand | <0.001 |

variables

**This is a lower bound of the true significance, as provided by SPSS*

***Abbreviations: BDNF=brain-derived neurotrophic factor, T25FW= 25-feet walk test, 9HPT= 9 holes peg test*

**Figure S1*.** Baseline BDNF differences between MS and HC

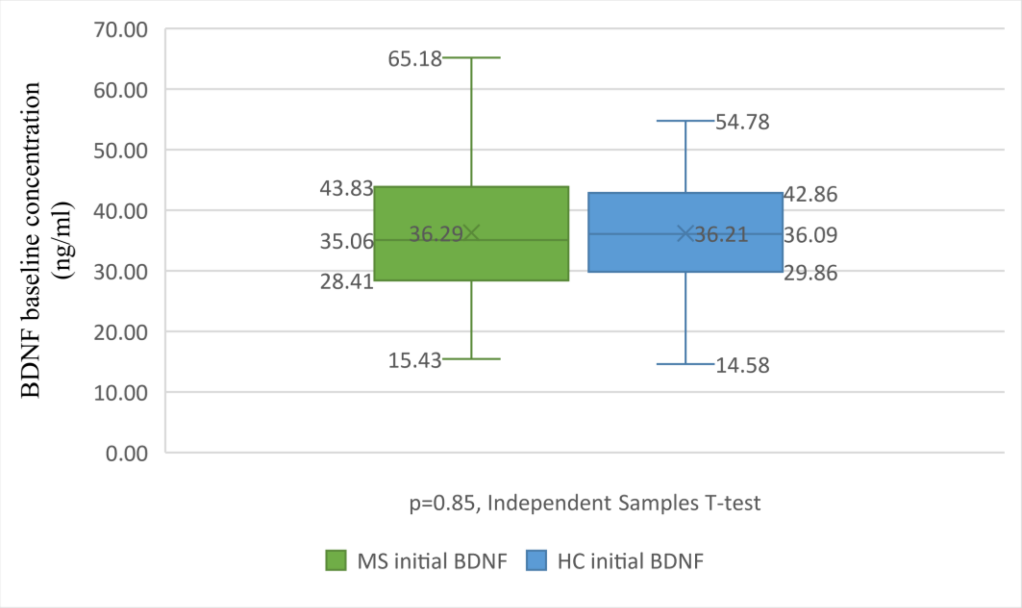


*Boxplot graph presenting the differences between baseline BDNF levels of MS patients (in green) and HC (in blue). Independent Samples T-Test reveals no statistically significant differences between the groups (p=0.85). BDNF, brain-derived neurotrophic factor; HC, healthy controls; MS, multiple sclerosis. X, mean value; Lower box limit, 25% quartile value; Central line, median value; Upper box limit, 75% quartile value; Lower whisker, minimum value; Upper whisker, maximum value.*

**Table S2*:** Mean and standard deviation of BDNF for Season as a fixed factor

| Season | Mean | SD |
|---|---|---|
| Warm | 23.51 | 8.841 |
| Cold | 22.27 | 10.044 |

**two-way ANOVA (Univariate Analysis of Variance)*

*SD=standard deviation*

**Table S3*:** Mean and standard deviation of BDNF for MS Type and gender as fixed factors and their combined influence

| MS type | Gender | Mean | SD |
|---|---|---|---|
| HC | Total | 16.74 | 7.142 |
| | Male | 10.41 | 3.885 |
| | Female | 19.62 | 6.427 |
| CIS | Total | 19.69 | 6.586 |
| | Male | 15.95 | 8.104 |
| | Female | 22.49 | 4.311 |
| RRMS | Total | 22.89 | 9.421 |
| | Male | 21.11 | 10.505 |
| | Female | 23.83 | 8.756 |

**two-way ANOVA (Univariate Analysis of Variances; SD=standard deviation*

**Table S4*:** Mean and standard deviation of BDNF for Treatment, background, and gender as fixed factors and their combined influence

| Treatment | Background | Gender | Mean | SD |
|---|---|---|---|---|
| HC | Total | Total | 16.75 | 7.143 |
| | | Male | 10.41 | 3.885 |
| | | Female | 19.62 | 6.428 |
| | Rural | Total | - | - |
| | | Male | - | - |
| | | Female | - | - |

| | | | | |
|---|---|---|---|---|
| | Urban | Total | 16.75 | 7.143 |
| | | Male | 10.41 | 3.885 |
| | | Female | 19.62 | 6.428 |
| Teriflunomide | Total | Total | 26.75 | 9.995 |
| | | Male | 24.10 | 9.174 |
| | | Female | 28.14 | 10.338 |
| | Rural | Total | 24.74 | 11.010 |
| | | Male | 27.66 | 10.497 |
| | | Female | 22.41 | 12.022 |
| | Urban | Total | 27.54 | 9.716 |
| | | Male | 22.07 | 8.480 |
| | | Female | 29.93 | 9.466 |
| Interferon beta1a | Total | Total | 22.06 | 8.030 |
| | | Male | 22.98 | 11.219 |
| | | Female | 21.52 | 5.756 |
| | Rural | Total | 18.69 | 7.126 |
| | | Male | 17.70 | 4.661 |
| | | Female | 19.49 | 9.137 |
| | Urban | Total | 23.50 | 8.119 |
| | | Male | 26.01 | 13.024 |
| | | Female | 22.24 | 4.246 |

**two-way ANOVA (Univariate Analysis of Variances; SD=standard deviation*

**Table S5*:** Mean and standard deviation of BDNF for Treatment, background, and season as fixed factors and their combined influence

| Treatment | Background | Season | Mean | SD |
|---|---|---|---|---|
| HC | Total | Total | 16.75 | 7.143 |
| | | Warm | - | - |

| | | | | |
|---|---|---|---|---|
| | | Cold | 16.75 | 7.143 |
| | Rural | Total | - | - |
| | | Warm | - | - |
| | | Cold | - | - |
| | Urban | Total | 16.75 | 7.143 |
| | | Warm | - | - |
| | | Cold | 16.75 | 7.143 |
| Teriflunomide | Total | Total | 26.75 | 9.995 |
| | | Warm | 25.14 | 10.082 |
| | | Cold | 30.31 | 9.300 |
| | Rural | Total | 24.74 | 11.010 |
| | | Warm | 20.18 | 8.173 |
| | | Cold | 30.44 | 12.500 |
| | Urban | Total | 27.54 | 9.716 |
| | | Warm | 26.59 | 10.335 |
| | | Cold | 30.22 | 7.868 |
| Interferon beta1a | Total | Total | 22.06 | 8.030 |
| | | Warm | 21.42 | 6.628 |
| | | Cold | 22.89 | 9.793 |
| | Rural | Total | 18.69 | 7.126 |
| | | Warm | 20.15 | 6.450 |
| | | Cold | 17.53 | 8.157 |
| | Urban | Total | 23.50 | 8.119 |
| | | Warm | 21.81 | 6.891 |
| | | Cold | 26.25 | 9.642 |

**two-way ANOVA (Univariate Analysis of Variances; SD=standard deviation*
